# Fabp5 Is the Key Regulator Mediating γ‐CEHC Differentiation in Osteoblasts and Osteoclasts

**DOI:** 10.1002/biof.70079

**Published:** 2026-01-19

**Authors:** Cheng Cheng, Rong Chen, Minjuan Li, Shuai Lu, Xinping Li, Gengli Cui, Hailing Chen, Xieyuan Jiang

**Affiliations:** ^1^ Department of Osteoporosis Beijing Jishuitan Hospital, Capital Medical University Beijing China; ^2^ Department of Orthopedic Trauma Beijing Jishuitan Hospital, Capital Medical University Beijing China; ^3^ Beijing Research Institute of Traumatology and Orthopaedics Beijing China

**Keywords:** Fabp5, macrophage polarization, osteoblast, osteoclast, osteoporosis, thermal proteome profiling, γ‐CEHC

## Abstract

Osteoporosis is closely linked to oxidative stress and inflammation, positioning the vitamin E metabolite γ‐CEHC, known for its robust antioxidant and anti‐inflammatory properties, as a promising therapeutic agent. However, its molecular targets have remained largely unknown. In this study, we characterized the protein targets of γ‐CEHC and clarified its role in regulating bone metabolism using an ovariectomized (OVX) mouse model and in vitro assays. Bone morphological analysis and histomorphometry demonstrated that γ‐CEHC improves osteoporosis in OVX mice by inhibiting osteoclast differentiation and enhancing osteoblast differentiation. To identify the underlying mechanisms, we employed isothermal thermal proteome profiling (TPP) to map γ‐CEHC‐interacting proteins, followed by Gene Ontology (GO) and KEGG enrichment analyses. Our findings identified fatty acid‐binding protein 5 (Fabp5) as a core target. The direct and specific binding between γ‐CEHC and Fabp5 was confirmed through cellular thermal shift assays (CETSA), molecular docking—suggesting hydrogen bonding with Thr63—and Surface Plasmon Resonance (SPR) which showed a strong binding affinity (Kd = 5.24 μM). Furthermore, γ‐CEHC was found to suppress LPS‐induced M1 macrophage activation and promote M2 polarization, thereby reducing reactive oxygen species (ROS) levels and restoring bone remodeling homeostasis. This study is the first to systematically elucidate the molecular mechanisms of γ‐CEHC in bone metabolism, revealing that it acts as a highly selective ligand for Fabp5. These findings provide a novel mechanistic basis for using γ‐CEHC and targeting Fabp5 in the treatment of osteoporosis.

## Graphical abstract

γ‐CEHC, as a natural Fabp5 inhibitor, ameliorates the osteoporotic microenvironment through a triple‐action mechanism—targeting Fabp5 inhibition → modulating macrophage polarization → balancing bone metabolism—highlighting its translational medical value.

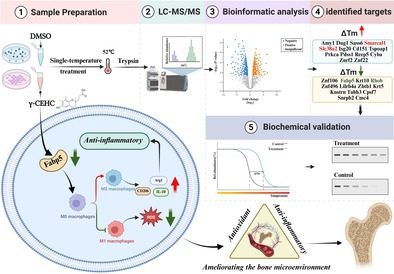

## 1 Introduction

Osteoporosis (OP), a systemic skeletal disorder defined by lowered bone mass and microarchitectural breakdown, causes bone fragility and fracture risk [1], representing a significant public health concern [2]. Bone loss progresses without noticeable clinical symptoms. When complications arise, the reduction in bone mass is often irreversible [3]. Previous research has shown that inflammation and oxidative stress are common in populations at risk for OP, including postmenopausal women, elderly men, and diabetic and obese patients. Targeting oxidative stress and inflammation could improve therapeutic outcomes for OP [4].

The vitamin E (VE) family includes eight lipophilic antioxidants, including α‐, β‐, γ‐, and δ‐tocopherol (αT, βT, γT, δT) as well as α‐, β‐, γ‐, and δ‐tocotrienol (αTE, βTE, γTE, δTE) [5]. VE is a potent antioxidant, particularly when scavenging ROS and free radicals [6]. Most studies on the bone‐protective effects of VE have focused on αT [7, 8, 9, 10, 11] and tocotrienols [12, 13, 14, 15]. However, mechanistic investigations using preclinical animal models have revealed that γT has characteristics absent in αT. Notably, γT undergoes more extensive metabolism than αT. Its metabolites, including 2,7,8‐trimethyl‐2(2′‐carboxyethyl)‐6‐hydroxychroman (γ‐CEHC) and 13′‐carboxychromanol (13′‐COOH), exert biological effects unlike their precursors [5], with improved anti‐inflammatory and antioxidant properties compared to unmetabolized forms [16]. Wechter et al. demonstrated that γ‐CEHC inhibits the 70 pS potassium channel in the kidney's thick ascending limb cells [17]. γ‐CEHC inhibits prostaglandin E2 (PGE2) synthesis in LPS‐stimulated RAW264.7 macrophages and IL‐1β‐treated A549 human epithelial cells by suppressing COX‐2 activity, with a half‐maximal inhibitory concentration (IC_50_) of 30 μM. In contrast, 50 μM α‐tocopherol (αT) lowered PGE2 production by only 25% in macrophages and did not affect epithelial cells. In COX‐2‐preinduced cells, γ‐CEHC suppressed PGE2 synthesis following arachidonic acid (AA) supplementation after 1 h of incubation. γ‐tocopherol (γT) required 8 to 24 h under the same conditions to exert similar inhibitory effects [18]. An open‐label study demonstrated that γT, α‐CEHC, and γ‐CEHC, but not αT, moderately inhibited LPS‐induced IκBα degradation in peripheral blood mononuclear cells (PBMCs). The same study demonstrated that γT, unaffected by αT, suppressed LPS‐induced JNK phosphorylation, while α‐CEHC and γ‐CEHC had differential inhibitory effects on JNK phosphorylation [19]. Our unpublished data showed significantly lower serum γ‐CEHC levels in osteoporotic patients compared to healthy controls. γ‐CEHC has potent anti‐inflammatory and antioxidant activities in chronic diseases such as OP; however, its protein targets and molecular mechanisms remain unexplored.

TPP is a global proteomics approach for detecting changes in protein thermal stability [20]. It is founded on the binding of small molecules, such as drugs or metabolites, that alter the thermal stability of their target proteins [21]. By integrating mass spectrometry, TPP assesses proteome‐wide shifts in stability across temperature gradients, enabling the identification of networks between compounds and proteins.

This study established an OVX mouse model and confirmed that γ‐CEHC exerts anti‐osteoporotic effects in vivo and in vitro by bidirectionally regulating bone metabolism. By employing TPP, we systematically mapped the γ‐CEHC interactome in osteoblastic MC3T3‐E1 and osteoclastic RAW264.7 cells, identifying 29 high‐confidence target proteins. The thermal stability changes and role of the core target Fabp5 were validated in murine and human cell lines. γ‐CEHC was found to function by specifically binding to Fabp5 and modulating its activity. Mechanistic studies demonstrated that γ‐CEHC significantly suppresses oxidative stress and promotes macrophage polarization from M1 to M2, restoring bone remodeling homeostasis.

We constructed a multidimensional evidence chain for “γ‐CEHC‐Fabp5‐macrophage polarization‐bone metabolism regulation”, comprehensively elucidating the novel anti‐osteoporotic mechanism by which γ‐CEHC specifically targets Fabp5, regulates macrophage M1/M2 phenotypic switching, and thereby bidirectionally regulates osteoblast/osteoclast differentiation. This study establishes a methodological paradigm for interpreting the multi‐level action mode of “target identification‐pathway regulation‐phenotypic validation” for natural active metabolites. Using γ‐CEHC, our research demonstrates that the integrated strategy combining TPP technology, multi‐species validation, and molecular interaction analysis holds significant scientific value for deciphering the functional mechanisms of endogenous metabolites in complex diseases.

## 2 Methods

### 2.1 Cell Lines, Antibodies, Compounds, and Reagents

MC3T3‐E1 subclone 14 is a cellular model for osteogenic differentiation, and RAW264.7 cells can differentiate into osteoclasts upon RANKL induction. These two murine cell lines were selected as experimental models in this study. To demonstrate the universality and clinical relevance of our findings, we employed hFOB1.19 cells and human monocytic/macrophage THP‐1 cells as experimental models. MC3T3‐E1 subclone 14 and RAW264.7 cells were obtained from Zhong Qiao Xin Zhou Biotechnology Co. Ltd. (Shanghai, China). The human osteoblast cell line hFOB1.19 and the human THP‐1 monocytic leukemia cell line were purchased from the Shanghai Cell Bank of the Chinese Academy of Medical Sciences (Shanghai, China). Primary antibodies were as follows: glyceraldehyde‐3‐phosphate dehydrogenase (GAPDH; Abcam, ab9485), Runt‐related transcription factor 2 (Runx2; Abcam, ab236639), osteocalcin (OCN; Abcam, ab133612), collagen I (Col1; Bioss, bs‐7158R), cathepsin K (Ctsk; Abcam, ab300569), FBJ murine osteosarcoma viral oncogene homolog (c‐Fos; Abcam, ab208942), nuclear factor of activated T‐cells, cytoplasmic 1 (Nfatc1; Cell Signaling Technology, #8032), SWI/SNF‐related, matrix‐associated, actin‐dependent regulator of chromatin subfamily A‐like 1 (Smarcal1; Sigma, ABE1836), Ras homolog family member B (Rhob; Cell Signaling Technology, #2098), and fatty acid‐binding protein 5 (Fabp5; Proteintech, 12348‐1‐AP). Horseradish peroxidase‐conjugated secondary antibodies were obtained from Agilent Technologies Inc., including γ‐CEHC and α‐CEHC (MedChemExpress, Monmouth Junction, NJ, USA) and dissolved in DMSO (Wako Pure Chemical Industries Ltd., Osaka, Japan). Media, including fetal bovine serum (FBS), α‐minimal essential medium (α‐MEM), Dulbecco's modified Eagle medium/Ham's F‐12 nutrient mixture (DMEM/F12), Roswell Park Memorial Institute 1640 medium (RPMI‐1640), and Dulbecco's modified Eagle's medium (DMEM), were obtained from Gibco BRL. β‐Glycerol disodium phosphate (G9422), ascorbic acid (A92902), phorbol‐12‐myristate‐13‐acetate (PMA, 524400), Macrophage colony‐stimulating factor (M‐CSF, M6518), and dexamethasone (D4902) were obtained from Sigma‐Aldrich. Recombinant mouse RANKL (CJ94) was obtained from NovoProtein Technology Co. Ltd. CM5 Sensor Chip (BR‐1005‐30), Amine Coupling Kit (BR100050), 10× phosphate‐buffered saline (PBS)‐*P*+ buffer (28995084), and Sodium acetate buffer (BR100349) were acquired from Cytiva.

### 2.2 In Vivo Study

#### 2.2.1 Mouse Model

12‐week‐old female C57BL/6J mice were obtained from Weitong Lihua Animal Technology. All mice were housed in specific pathogen‐free (SPF) facilities at Beijing Jishuitan Hospital, Capital Medical University, at 20°C–24°C and 40%–60% relative humidity with a 12‐h light/dark cycle in group cages. The animals were provided with a standard chow diet and water ad libitum. During surgical procedures, mice were anesthetized by intramuscular injection of Ketamine Hydrochloride and Domitor, followed by operative site shaving. A 1 cm incision was made through the skin and peritoneum, approximately 1 cm lateral to the spinal column. In the model group, bilateral ovary excision was performed after ligation, whereas in the control group, only small adipose tissue deposits were removed. The incisions were sutured and disinfected with iodine. Drug treatment began 1 week after OVX.

Wild‐type mice were separated into: sham operation, OVX model, OVX + γ‐CEHC (5 mg/kg), and OVX + γ‐CEHC (15 mg/kg) groups (*n* = 6 per group). γ‐CEHC was dissolved in dimethyl sulfoxide and diluted in double‐distilled water containing 0.03% Tween‐80. The treatment was administered by oral gavage five times weekly for 6 weeks. Euthanasia was conducted by cervical dislocation. Femurs were obtained for histomorphometric analysis. All animal housing and experimental procedures in this study were performed in accordance with the protocol, which was reviewed and approved by the Animal Ethics Committee of Beijing Jishuitan Hospital, Capital Medical University (No. 2025‐11‐10), and complied with the ethical standards established by the Institutional Animal Care and Use Committee.

#### 2.2.2 Micro‐CT Analysis

Fixed, non‐decalcified mouse left femurs were subjected to micro‐CT analysis using a scan voltage of 80 kV, current of 100 μA, and an isotropic voxel size of 8 μm. Following scanning, trabecular bone measurements were conducted within a 1.5‐mm‐long region of interest (ROI) beginning 0.5 mm proximal to the distal growth plate. The trabecular parameters included bone volume fraction, trabecular number, trabecular thickness, and trabecular separation. All data extraction and reconstruction were conducted using the manufacturer's proprietary analysis software.

#### 2.2.3 Tartrate‐Resistant Acid Phosphatase (TRAP) Staining

The right femurs of mice were decalcified in 10% EDTA solution for 2 weeks, then embedded in paraffin and sectioned at 5 μm. Osteoclast formation was evaluated by TRAP staining.

#### 2.2.4 ELISA of Serum PINP and CXT‐1

Serum samples were collected from the mouse blood. Levels of serum P1NP and CTX‐1 were quantified using specific ELISA kits, including a PINP ELISA kit (Meilian Biotechnology, Shanghai, ml038002) and a CTX‐1 ELISA kit (Bangyi Biotechnology, Shanghai, BYE30118), following the manufacturers' instructions.

### 2.3 In Vitro Study

#### 2.3.1 Cell Culture

RAW264.7 cells were grown in DMEM supplemented with 10% FBS and 1% penicillin–streptomycin. MC3T3‐E1 cells were maintained in α‐MEM containing 10% FBS and 1% penicillin–streptomycin. THP‐1 cells were grown in RPMI‐1640 medium containing 10% FBS, 0.05 mM β‐mercaptoethanol, and 1% PBS. Both cell lines were incubated at 37°C in a humidified atmosphere with 5% CO_2_. hFOB1.19 cells were maintained in DMEM/F12 medium supplemented with 10% FBS and 1% penicillin–streptomycin in incubators at 33.5°C, with 5% CO_2_.

RAW264.7 cells were stimulated with RANKL for 3 days to induce osteoclast differentiation. MC3T3‐E1 cells were treated with 100 nM dexamethasone, 10 mM β‐glycerophosphate, and 50 μM ascorbic acid for 14–21 days to cause osteoblast differentiation. THP‐1 cells were differentiated into macrophages using 200 ng/mL PMA, followed by 14‐day induction of osteoclast‐like identity with 25 ng/mL M‐CSF and 30 ng/mL RANKL. hFOB1.19 cells were treated with 100 nM dexamethasone, 10 mM β‐glycerophosphate, and 50 μM ascorbic acid while shifting the incubator temperature from 33.5°C to 39.5°C for 14–21 days to induce osteogenic differentiation. Cells were harvested, and pellets were stored at −80°C for subsequent analysis.

#### 2.3.2 Cytotoxicity Characterization

RAW264.7 cells or MC3T3‐E1 cells were grown with varying concentrations (0–60 μM) of γ‐CEHC for 24 h. Cell viability was subsequently assessed using a CCK‐8 assay kit according to the manufacturer's instructions.

#### 2.3.3 Quantitative Real‐Time Polymerase Chain Reaction (qRT–PCR)

We extracted RNA from RAW264.7 cells and MC3T3‐E1 cells using an RNAprep Pure Tissue Kit or Cell/Bacteria Kit (Tiangen, Beijing, China). mRNA and cDNA were quantified using TransScript cDNA Synthesis SuperMix (Transgen). Quantitative real‐time PCR (qRT‐PCR) was performed using TransStart Top Green qPCR SuperMix (Transgen). We determined relative gene expression using the ΔΔCq method. Primers are provided in Supplementary Table S1.

#### 2.3.4 Western Blotting

We collected cells in RIPA lysis buffer (Beyotime) with protease and phosphatase inhibitor cocktails. Protein concentrations were determined with a BCA assay. After adding 5× loading buffer, proteins were separated using SDS‐PAGE and transferred to PVDF membranes (Immobilon‐P, Millipore). We blocked membranes for 1 h at room temperature with 5% milk in Tris‐buffered saline containing 0.1% Tween‐20 (TBST). Membranes were incubated with primary antibodies at 4°C for at least 12 h. After washing membranes three times with TBST for 30 min each, we incubated them with horseradish peroxidase‐conjugated anti‐rabbit IgG or anti‐mouse IgG antibodies (both at 1:5000) at room temperature for 1 h. Immunoblot detection was conducted using ECL (Thermo Fisher Scientific).

#### 2.3.5 Isothermal Proteome Profiling Analysis

Cellular thermal shift assays were performed as previously described [20]. Cryopreserved cells at −80°C were thawed and resuspended in lysis buffer (PBS containing 1% protease inhibitor cocktail). Homogenized cells underwent three freeze–thaw cycles: flash‐freezing in liquid nitrogen for 2 min, followed by thawing at 37°C for 2 min, until complete lysis was achieved. After the final thaw, the lysate was centrifuged at 20,000 × *g* for 10 min at 4°C, and the supernatant was transferred to fresh centrifuge tubes. Protein concentrations were determined using a BCA protein assay kit according to the manufacturer's protocol. Each protein sample was separated into two equal aliquots. Each aliquot was treated with 30 μM γ‐CEHC or an equivalent volume of DMSO, then incubated at room temperature for 20 min. Heat treatment was administered at 52°C for 3 min, and equal volumes of supernatant were collected after centrifugation for liquid chromatography–tandem mass spectrometry (LC–MS/MS) analysis.

#### 2.3.6 LC–MS/MS Analysis

Digested peptides were dissolved in solvent A and loaded onto a homemade reversed‐phase analytical column (25 cm length, 100 μm inner diameter). The mobile phases consisted of solvent A (0.1% formic acid, 2% acetonitrile in water) and solvent B (0.1% formic acid in acetonitrile). Peptides were separated using the following gradient at a constant flow rate of 450 nL/min on a NanoElute UHPLC system (Bruker Daltonics): 0–40 min, 6% to 24% B; 40–52 min, 24% to 35% B; 52–56 min, 35% to 80% B; 56–60 min, 80% B. Peptides entered the capillary source and were analyzed using a timsTOF Pro 6 mass spectrometer. An electrospray voltage of 1.75 kV was applied. The TOF detector recorded precursor and fragment ions. The timsTOF Pro operated in data‐independent parallel accumulation–serial fragmentation (dia‐PASEF) mode. The full MS scan range was 100–1700 m/z (MS/MS scan range) and 16 PASEF MS/MS scans were acquired per cycle. The MS/MS scan range was 400–1200 m/z, with a window of 25 m/z.

#### 2.3.7 Database Search

DIA data were processed using Spectronaut (v.18) software. Tandem mass spectra were compared against the Mus_musculus_10090_SP_20231220.fasta database (17,191 entries) and concatenated with a reverse‐decoy database. We specified Trypsin/P as the cleavage enzyme, allowing up to two mismatched cleavages. Carbamidomethylation of cysteine was a fixed modification, whereas acetylation at the protein N‐terminus and oxidation of methionine were variable. We controlled the false discovery rate (FDR) at 1%.

#### 2.3.8 Bioinformatic Analysis

GO annotation was performed using the EggNOG‐mapper to assign GO terms to identified proteins based on the EggNOG database. We classified and annotated these proteins based on cellular components, molecular functions, and biological processes. Protein domains were annotated using the Pfam database and the PfamScan tool. Protein pathway annotation relied on the KEGG pathway database, with protein identification performed using BLAST alignment (blastp, e‐value ≤ 1e‐4). For each sequence, annotations were based on the highest‐scoring alignment result. Fisher's exact test was applied to identify significantly enriched functions among differentially expressed proteins. Functional terms with fold enrichment over 1.5 and *p*‐values less than 0.05 were significantly enriched.

#### 2.3.9 Cellular Thermal Shift Assay (CETSA)

The Western blotting (WB)‐CETSA experiment was performed according to a previously described method [22]. Cell lysates were incubated with either γ‐CEHC or DMSO at room temperature for 20 min. The lysates were separated into six equal aliquots and heated at six temperature points (37°C, 42°C, 47°C, 52°C, 57°C, 62°C, and 67°C) for 3 min. After centrifugation, equal volumes of the supernatant were used for Western blot analysis.

#### 2.3.10 Molecular Docking

The AlphaFold Fabp5 (UniProt ID: Q05816) file was downloaded from the UniProt database (https://www.uniprot.org/), while the 3D SDF structure of the small molecule γ‐CEHC (CHEBI:89379) was obtained from the PubChem database (https://www.ebi.ac.uk/chebi/searchId.do?chebiId=CHEBI:89379). Molecular docking between Fabp5 and γ‐CEHC was conducted using AutoDock Vina software (version 1.5.7) [23] with default parameters. The binding affinity was characterized by the binding energy between the two molecules; a value below −7.0 kcal/mol typically indicates strong binding affinity. The three‐dimensional binding model was constructed using PyMOL (https://pymol.org/).

#### 2.3.11 Surface Plasmon Resonance (SPR)

Experiments were performed at 25°C on a BIAcore 1K using CM5 sensor chips, and data were analyzed using BIAcore 1K Evaluation software (Cytiva) following the manufacturer's instructions. A cell on the CM5 sensor chip was activated with a mixture of 200 μM 1‐ethyl‐3‐(3‐dimethylaminopropyl)carbodiimide (EDC) and 50 μM N‐hydroxysuccinimide (NHS) at 10 μL min^−1^ for 420 s. A total of 50 μL of Fabp5 protein, mixed with 180 μL of 10 mM sodium acetate solution, pH 5.0, was immobilized on the surface of the cell at 10 μL min^−1^ for 420 s for two repetitive runs. The cell was then blocked with 1 M ethanolamine (10 μL min^−1^ for 420 s). A neighboring lane serving as a reference was similarly activated and blocked, with PBS adjusted to pH 5.0 for immobilization. Both lanes were equilibrated with PBS. The stock solution was diluted to a series of concentrations in PBS and flowed at 10 μL min^−1^ for 150 s. At the end of each flow, cells were regenerated for 5 min with 10 mM glycine‐HCl (pH 2.0) solution at 10 μL min^−1^. Data from the sample cell were collected using Biacore Insight (v.2.0, Cytiva) and were subtracted from those from the reference cell. Association and dissociation constants were determined by global fitting of the data to a 1:1 Langmuir binding model using BIAcore 1K Evaluation software (Cytiva, Marlborough, MA, USA). Data were exported to Origin 7 software (v.7.0552, OriginLab) to produce final figures.

#### 2.3.12 Macrophage Polarization and γ‐CEHC Treatment

We seeded MC3T3‐E1 and RAW264.7 cells at a density of 2.5 × 10^5^ cells per well in 6‐well plates and cultured them for 24 h. To induce macrophage polarization, we treated RAW264.7 cells with 1 μg/mL LPS (MedChemExpress) for 24 h. Following M1 polarization, we treated the cells with 30 μM γ‐CEHC for 24 h.

#### 2.3.13 Small Interfering RNA (siRNA) Synthesis and Transfection

siRNA targeting *Fabp5* and the corresponding negative control were synthesized by GeneChem (Shanghai, China). Following the manufacturer's protocol, RAW264.7 cells were transfected with Lipofectamine 3000 (Invitrogen, Cat. No. L3000015). The *Fabp5* siRNA sequences used were as follows: siFABP5‐forward: 5′‐UGGGAAGGAAAGCACAAUU‐3′ and siFABP5‐reverse: 5′‐UAUUGCUUUCCUUCCCAUU‐3′. Cells transfected with negative control siRNA and FABP5 siRNA were si‐NC and si‐Fabp5 groups, respectively.

#### 2.3.14 Intracellular ROS Detection

A Reactive Oxygen Species Assay kit (Beyotime Institute of Biotechnology) was used to assess ROS production, according to the manufacturer's protocol. A flow cytometer was used to monitor production, and the results were analyzed using FlowJo (v.10.9.0).

### 2.4 Statistical Analysis

All statistical analyses were conducted using GraphPad Prism 9.0. Each experiment was independently repeated in triplicate. Data are mean ± standard error of the mean (SEM). Unpaired Student's *t*‐tests were used for comparisons between two groups, while one‐way or two‐way analysis of variance (ANOVA) was applied for comparisons among three or more groups. A *p*‐value of less than 0.05 was statistically significant.

## 3 Results

### 3.1 γ‐CEHC Improves OVX‐Induced Osteoporosis

**FIGURE 1:**
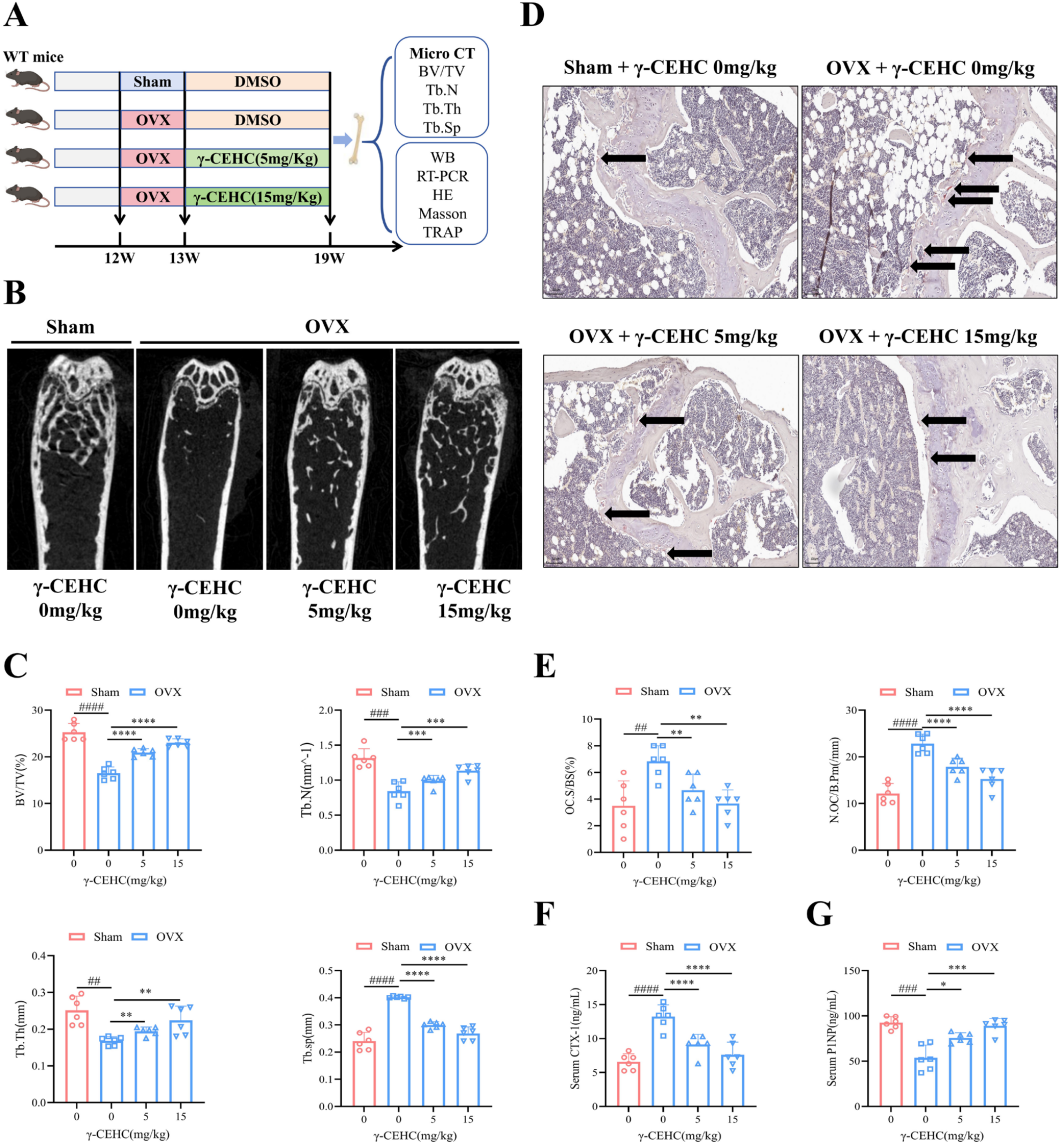
γ‐CEHC alleviates bone loss in OVX mice. (A) Administration of γ‐CEHC treatment in OVX mice. (B) Trabecular bone morphology of the femur in mice. (C) Analysis of trabecular morphological parameters, including BV/TV, Tb.N, Tb.Th, and Tb.Sp (*n* = 6). (D) Images of TRAP staining (Magnification, 100×) of rat femoral tissue, with black arrows indicating osteoclasts. (E) Histomorphometric analysis of osteoclasts, including N.Oc/B.Pm and Oc.S/BS (*n* = 6). (F) Plasma levels of the bone resorption marker CTX‐1 in mice (*n* = 6). (G) Plasma levels of the bone formation marker P1NP in mice (*n* = 6). Data were presented as means ± SD (^*#*^ *p* < 0.05, ^*##*^ *p* < 0.01, ^*###*^ *p* < 0.001 indicate statistically significant differences between the OVX group compared to the Sham group; *p < 0.05, **p < 0.01, ***p < 0.001 indicate statistically significant differences between the different concentrations of γ‐CEHC treatment groups versus the OVX group).

We investigated the effect of γ‐CEHC on the bone mass of OVX mice via oral gavage (Figure 1A). Selection of 5 and 15 mg/kg was based on the well‐established body surface area normalization method, with corrections applied to the doses reported in acute models [24, 25]. Trabecular bone morphological results demonstrated that OVX mice exhibited a significant reduction in trabecular bone in the distal femur compared to Sham‐operated mice. Both low‐dose and high‐dose γ‐CEHC treatment significantly increased trabecular bone volume in OVX mice (Figure 1B). Bone morphological parameters revealed that BV/TV, Tb.N, and Tb.Th were significantly decreased, while Tb.Sp was significantly elevated in OVX mice relative to Sham mice. Following treatment with low‐dose or high‐dose γ‐CEHC, BV/TV, Tb.N, and Tb.Th were significantly increased, while Tb.Sp was decreased (Figure 1C), suggesting an increase in trabecular bone number and density. These results indicate that γ‐CEHC alleviates bone loss in OVX mice.

TRAP staining of mouse femoral tissue showed a significant increase in osteoclast formation in OVX mice. In contrast, osteoclast formation was suppressed in the γ‐CEHC‐treated groups (Figure 1D). Bone histomorphometry demonstrated that γ‐CEHC treatment significantly reduced N.Oc/B.Pm and Oc.S/BS (Figure 1E). Additionally, plasma levels of the bone resorption marker CTX‐1 were significantly lower in the γ‐CEHC‐treated group relative to the control group (Figure 1F). In contrast, the bone formation marker P1NP was significantly higher (Figure 1G). These findings indicate that γ‐CEHC treatment inhibits osteoclast‐mediated bone resorption while promoting osteoblast‐mediated bone formation.

### 3.2 γ‐CEHC Promotes Osteoblast Differentiation and Inhibits Osteoclast Differentiation

**FIGURE 2:**
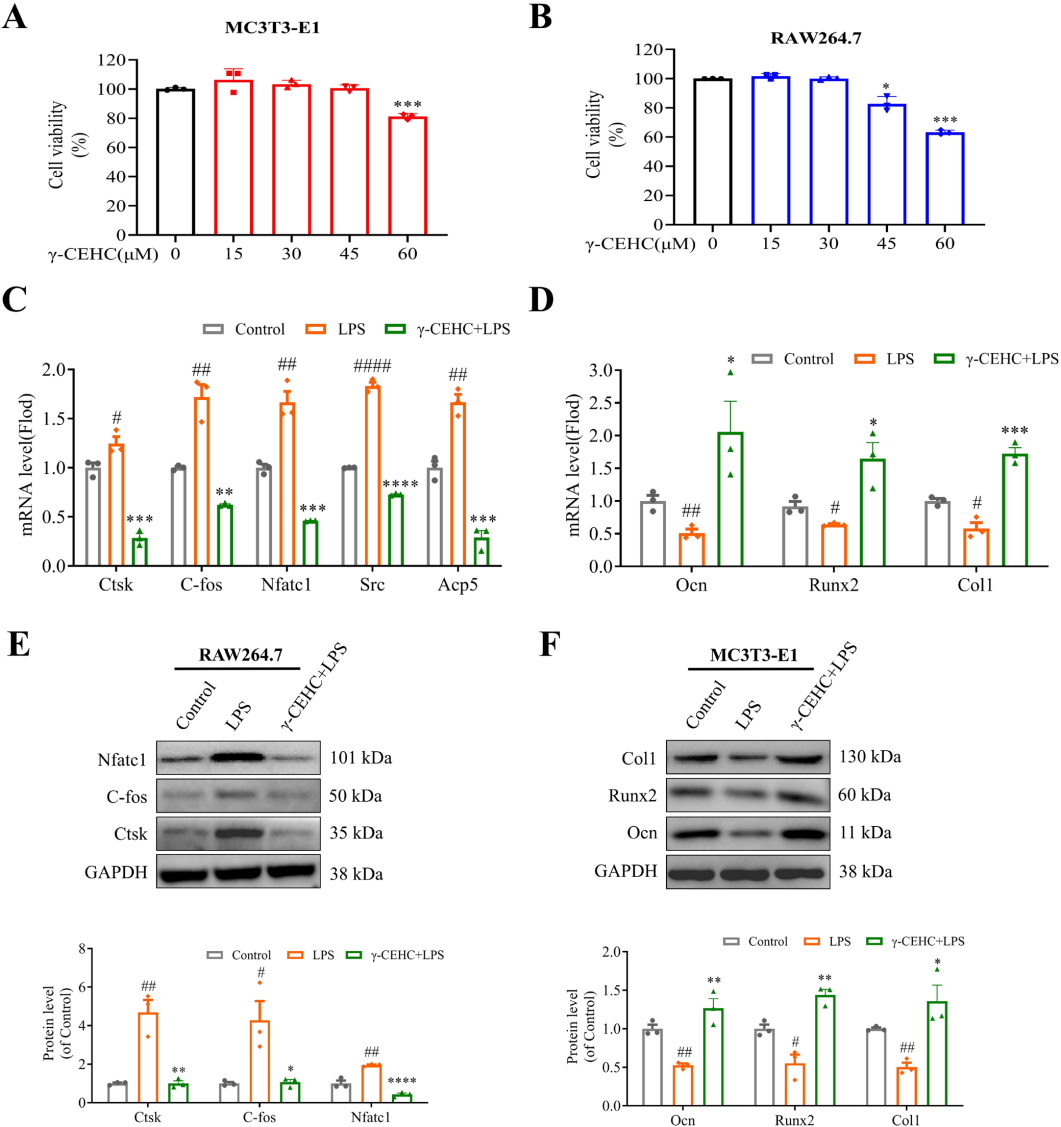
Effects of γ‐CEHC on osteoblast and osteoclast differentiation. (A) Cell viability of MC3T3‐E1 cells following 24‐h co‐culture with γ‐CEHC, determined by CCK‐8 assay. (B) Cell viability of RAW264.7 cells after a 24‐h co‐culture with γ‐CEHC, determined via CCK‐8 assay. (C) Expression of osteoclast marker genes (*Ctsk*, *C‐fos*, *Nfatc1*, *Scr*, and *Acp5*) detected by qRT‐PCR. (D) Expression of osteoblast marker genes (*Ocn*, *Runx2*, and *Col1*) detected by qRT‐PCR. (E) Protein expression levels of osteoclast markers (Ctsk, C‐fos, and Nfatc1) analyzed via Western blot. (F) Protein expression levels of osteoblast markers (Ocn, Runx2, and Col1) analyzed by Western blot. All measurements are presented as means ± SD for three biological replicates (^#^ *p* < 0.05, ^##^ *p* < 0.0*1*, ^###^ *p* < 0.001 indicate statistically significant differences between the LPS‐alone group versus the control group; *p < 0.05, **p < 0.01, ***p < 0.001 indicate statistically significant differences between the γ‐CEHC treatment group versus the LPS‐alone group).

In MC3T3‐E1 cells, cell viability assays demonstrated no significant cytotoxicity at concentrations under 60 μM (Figure 2A). In RAW264.7 cells, no significant cytotoxicity was found at concentrations below 45 μM (Figure 2B). Based on previous reports that γ‐CEHC inhibits LPS‐induced COX‐2 activity at 30 μM, we selected 30 μM to investigate the effects of γ‐CEHC on osteoblast and osteoclast differentiation. RT‐qPCR analysis of the impact of γ‐CEHC on osteoblast and osteoclast marker expression revealed that γ‐CEHC treatment significantly downregulated mRNA expression of *Ctsk*, *C‐fos*, *Nfatc1*, *Scr*, and *Acp5* genes (Figure 2C), while upregulating mRNA expression of *Ocn*, *Runx2*, and *Col1* genes (Figure 2D). Western blotting validated these gene expression patterns, supporting the dual regulatory role of γ‐CEHC in bone remodeling (Figure 2E,F).

In conclusion, γ‐CEHC exhibits dual efficacy, promoting bone formation and inhibiting bone resorption, highlighting its potential as a therapeutic agent for OP.

### 3.3 TPP Combined With Quantitative Proteomics to Identify γ‐CEHC‐Interacting Proteins

Both cellular and animal experimental results demonstrated the therapeutic potential of γ‐CEHC for OP, prompting further investigation into its mechanisms of action. We employed isothermal TPP combined with quantitative proteomics to map the potential target landscape underlying γ‐CEHC‐mediated inhibition of osteoclast differentiation and promotion of osteoblast differentiation. The experimental workflow is presented in Figure S1.

**FIGURE 3:**
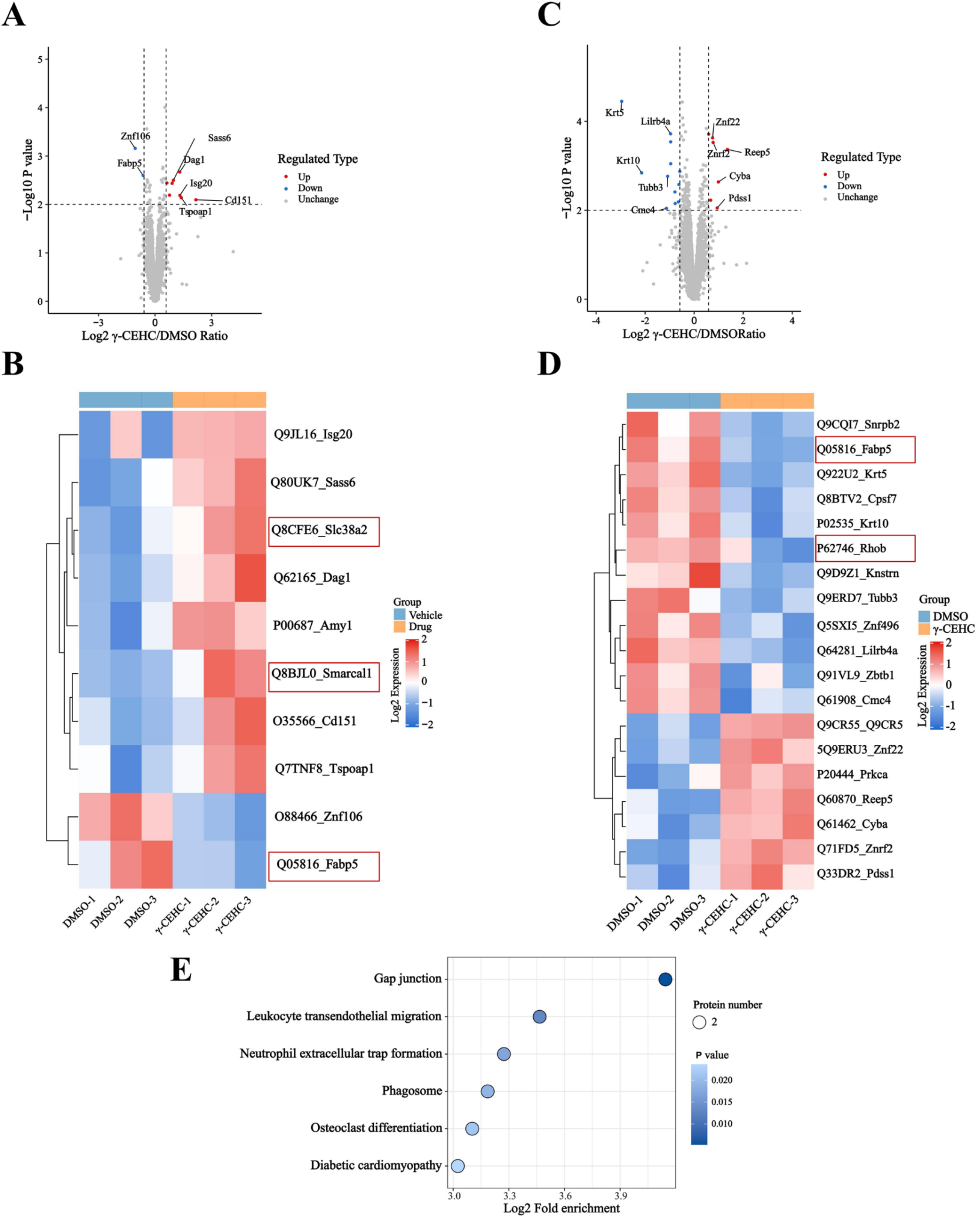
Isothermal TPP and label‐free quantitative proteomics reveal target profiles of γ‐CEHC intervention throughout osteoblast and osteoclast differentiation. (A, B) Volcano plot (A) and heatmap (B) of binding targets identified by isothermal TPP in MC3T3‐E1 cells. (C, D) Volcano plot (C) and heatmap (D) of binding targets identified by isothermal TPP in RAW264.7 cells. (E) KEGG pathway enrichment analysis of binding targets identified via isothermal TPP in RAW264.7 cells.

In MC3T3‐E1 cells, quantitative proteomic analysis using LC–MS/MS found 6401 proteins, of which 6400 were quantifiable (Figure S2A, Table S2). Quality control metrics, including peptide length distribution, molecular weight distribution, and protein coverage, validated dataset robustness (Figure S2B–D). Following γ‐CEHC treatment, the thermal stability of eight proteins significantly increased (|log_2_(fold change)|> 1.5, *p* < 0.01), while two proteins demonstrated reduced thermal stability (log_2_(fold change) < 0.66, *p* < 0.01) (Figure 3A, Table S3). Nearly half of these proteins are functionally linked to osteoblast differentiation and bone formation (Figure 3B).

To assess the biological functions of the 10 high‐confidence γ‐CEHC targets, we performed bioinformatic analyses of molecular functions, subcellular localization, and pathway involvement. GO enrichment analysis identified monocarboxylic acid transport, carboxylic acid transport, negative regulation of phosphorus metabolic processes, and negative regulation of phosphate metabolism. Therefore, γ‐CEHC may exert antioxidant effects by altering the availability of monocarboxylic acid substrates, indirectly modulating tricarboxylic acid (TCA) cycle flux and redox balance. Mechanistically, this may involve the suppression of phosphorylation‐dependent signaling pathways, such as the PI3K/AKT pathway, thereby reducing phosphatase activity. These targets are localized to the basement membrane, sarcolemma, and collagen‐containing extracellular matrix. Molecular function analysis demonstrated enrichment in DNA‐related catalytic activity and signaling receptor binding. γ‐CEHC may engage extracellular signals, including inflammatory mediators or hormones, via receptor interactions, activating DNA repair or epigenetic reprogramming pathways to coordinate cellular stress responses and maintain metabolic homeostasis (Figure S2E). KEGG pathway analysis did not identify any significantly enriched signaling pathways. However, γ‐CEHC target proteins are localized at the basement membrane, suppress phosphatase activity, and exhibit anti‐inflammatory and antioxidant effects.

In RAW264.7 cells, quantitative proteomic analysis using LC–MS/MS identified 6351 proteins, of which 6348 were quantifiable (Figure S3A, Table S4). Peptide length distribution, molecular weight distribution, and protein coverage confirmed the reliability and robustness of the dataset (Figure S3B–D). Following γ‐CEHC treatment, a significant increase in the thermal stability of 7 proteins (|log_2_(fold change)|> 1.5, *p* < 0.01) was observed, and a decrease in the thermal stability of 12 proteins (log_2_(fold change) < 0.66, *p* < 0.01) (Figure 3C, Table S5). Fabp5 was identified among the proteins with significantly reduced thermal stability, while Rhob exhibited a marked decrease in thermal stability (Figure 3D).

To characterize these effects, we performed GO classification and KEGG pathway enrichment analyses for 19 high‐confidence γ‐CEHC targets. GO analysis revealed that the top biological processes included responses to mineralocorticoids, positive regulation of endothelial cell proliferation, enhancement of inflammatory responses, promotion of lymphocyte differentiation, and regulation of T cell differentiation. γ‐CEHC target proteins play key roles in regulating endothelial and lymphocyte differentiation and modulating inflammatory pathways, aligning with previously reported biological functions. Cellular component analysis suggested that γ‐CEHC target proteins were localized to keratin filaments, intermediate filaments, polymeric cytoskeletal fibers, the intermediate filament cytoskeleton, and the membrane. This distribution suggests a distinct mechanism by which γ‐CEHC coordinates inflammatory signaling and maintains cellular homeostasis via structure–function coupling. Molecular function enrichment indicated that these proteins are associated with scaffold protein binding, cytoskeletal structural support, protein heterodimerization, signaling receptor activity, and transduction.

These findings suggest that γ‐CEHC may establish an integrated regulatory network encompassing multiple functional modules, ranging from scaffold assembly and cytoskeletal stabilization to receptor modulation and downstream signal transduction (Figure S3E). KEGG pathway enrichment analysis identified several pathways that were significantly enriched, including gap junctions, leukocyte *trans*‐endothelial migration, neutrophil extracellular trap formation, phagosome formation, osteoclast differentiation, and diabetic cardiomyopathy (Figure 3E). These findings indicate that γ‐CEHC may exert anti‐inflammatory and antioxidant effects through multiple mechanisms to mitigate OP: (1) by modulating connexin expression to influence signaling between macrophages and endothelial cells; (2) by inhibiting integrin activity while enhancing phagocytic efficiency; and (3) directly regulating osteoclast differentiation. Together, these pathways support the therapeutic potential of γ‐CEHC in treating OP.

### 3.4 Biochemical Validation of Identified γ‐CEHC Targets

**FIGURE 4:**
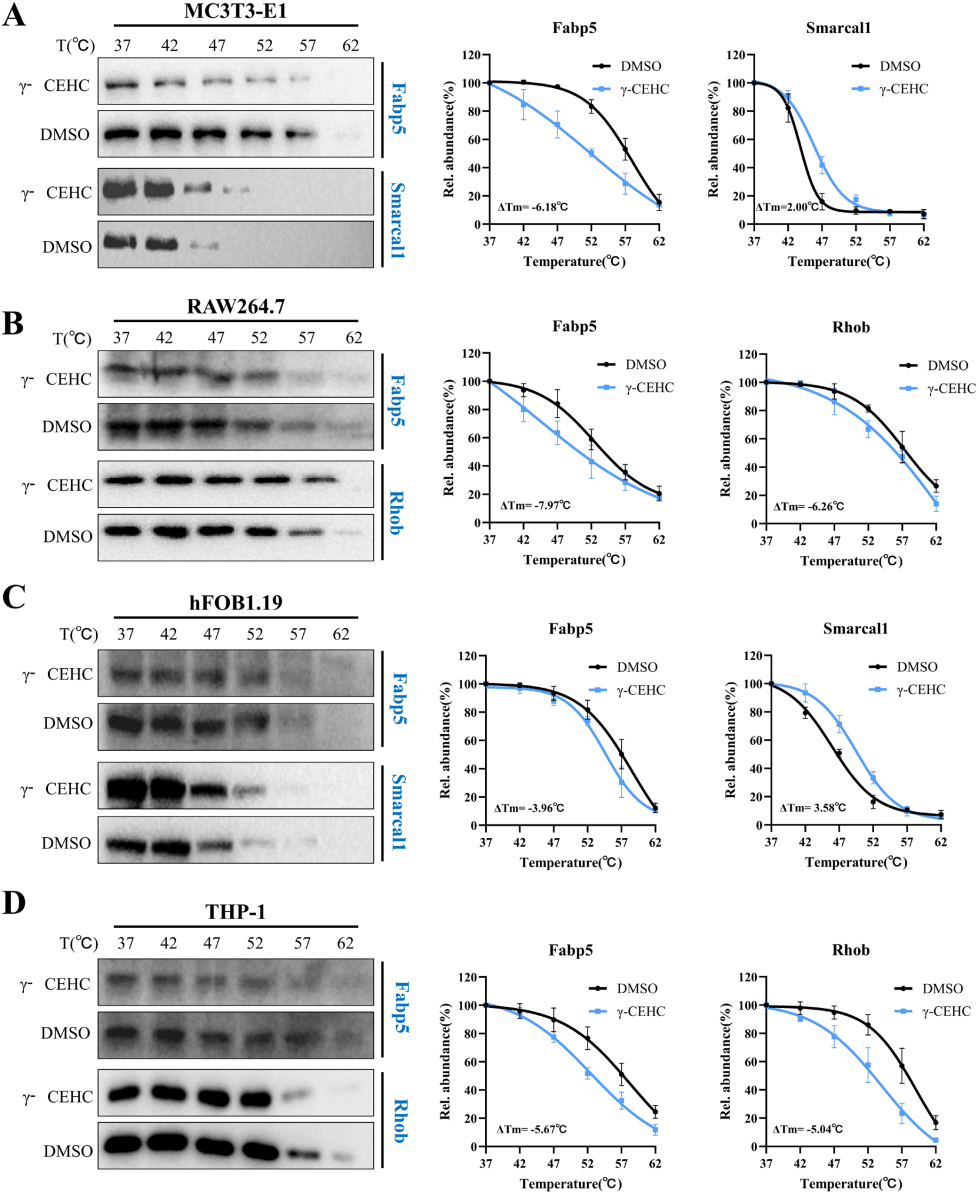
Validation of γ‐CEHC targets. (A) Thermal shift curves of γ‐CEHC targets (including Smarcal1 and Fabp5) in MC3T3‐E1 cells. (B) Thermal shift curves of γ‐CEHC targets (including Rhob and Fabp5) in RAW264.7 cells. (C) Thermal shift curves of γ‐CEHC targets (including Smarcal1 and Fabp5) in hFOB1.19 cells. (D) Thermal shift curves of γ‐CEHC targets (including Rhob and Fabp5) in THP‐1 cells. Blue curves are the γ‐CEHC‐treated group, while black curves are the DMSO‐treated group (control). Data are presented as means ± SD (*n* = 3).

Using protein differential change fold and the number of unique peptides identified, alongside a comprehensive consideration of the biological context of the targets, we selected Smarcal1 and Fabp5 for validation in MC3T3‐E1 cells, while Rhob and Fabp5 were chosen for RAW264.7 cells. We validated key γ‐CEHC targets using CETSA in lysates from osteogenically differentiated MC3T3‐E1 cells and osteoclastically differentiated RAW264.7 cells. In MC3T3‐E1 cells, γ‐CEHC increased the thermal stability of Smarcal1 (ΔTm = +2.00°C) and reduced the thermal stability of Fabp5 (ΔTm = −6.18°C) (Figure 4A). In RAW264.7 cells, γ‐CEHC similarly reduced the thermal stability of Fabp5 (ΔTm = −7.97°C) and Rhob (ΔTm = −6.26°C) (Figure 4B).

To confirm the universality and clinical relevance of these findings, we validated γ‐CEHC's key targets in lysates from human osteogenically differentiated hFOB1.19 cells and osteoclastically differentiated THP‐1 cells. In hFOB1.19 cells, γ‐CEHC increased the thermal stability of Smarcal1 (ΔTm = +3.58°C) and lowered the thermal stability of Fabp5 (ΔTm = −3.96°C) (Figure 4C). In THP‐1 cells, γ‐CEHC similarly reduced the thermal stability of both Fabp5 (ΔTm = −5.67°C) and Rhob (ΔTm = −5.04°C) (Figure 4D).

These results confirm that γ‐CEHC alters the thermal stability of its binding partners and validate the reliability of our TPP approach for mapping the γ‐CEHC interactome. Given that Fabp5 exhibited lowered stability across all cell types and is evolutionarily conserved between species, we propose that γ‐CEHC binds to Fabp5, causes conformational changes, downregulates Fabp5 protein expression, and modulates osteoblast and osteoclast differentiation.

### 3.5 The Specific Binding and Interaction Between γ‐CEHC and Fabp5

**FIGURE 5:**
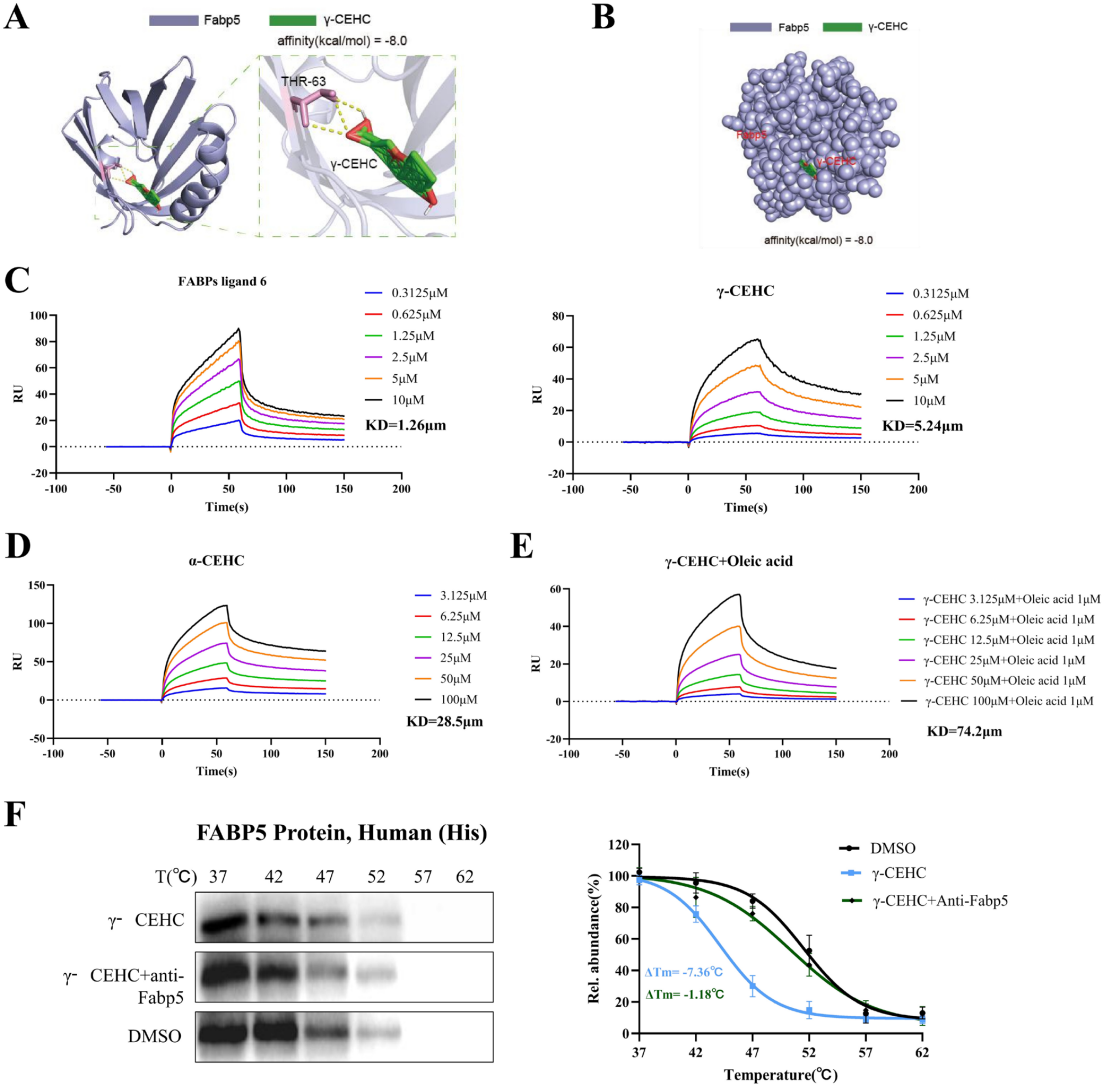
Molecular docking and SPR confirm the specificity of γ‐CEHC binding to Fabp5. (A) Molecular docking indicating the binding of γ‐CEHC to the β‐barrel domain of Fabp5. (B) Three‐dimensional simulation and binding energy of γ‐CEHC and Fabp5 interaction. (C) SPR sensorgrams indicate interactions between γ‐CEHC concentration gradients and FABP ligand 6 protein (left panel) versus Fabp5 protein (right panel). (D) SPR sensorgrams demonstrate the interaction between α‐CEHC concentration gradients and Fabp5 protein. (E) SPR sensorgrams demonstrate the interaction between γ‐CEHC concentration gradients and Fabp5 protein following treatment with oleic acid. (F) CETSA competition assays display thermal shift curves of Fabp5. Blue curves indicate the γ‐CEHC‐treated group, green curves indicate the γ‐CEHC + anti‐Fabp5 antibody group, while black curves represent the DMSO‐treated group (control). The green ΔTm values represent the ΔTm of the γ‐CEHC + anti‐Fabp5 antibody group relative to the DMSO‐treated group. In contrast, the blue ΔTm values represent the ΔTm of the γ‐CEHC group relative to the DMSO‐treated group.

Based on the hypothesis above, we used AutoDock Vina (v.1.5.7) to perform molecular docking between Fabp5 and γ‐CEHC to investigate their potential interaction. The docking results revealed that γ‐CEHC forms a hydrogen bond with the amino acid residue Thr63 of Fabp5 (Figure 5A). 3D simulation demonstrated that γ‐CEHC precisely binds to the β‐barrel of Fabp5, which is the canonical binding pocket for Fabp5 inhibitors [26], at −8 kcal/mol (Figure 5B). This indicates that γ‐CEHC is a natural inhibitor that exhibits strong spontaneous binding affinity for Fabp5. To validate that γ‐CEHC indeed binds to Fabp5, we performed SPR experiments. Previous studies indicate that FABP ligand 6 is a Fabp5 inhibitor with a dissociation constant (K_d_) of 0.874 μM [27]. Thus, we selected FABP ligand 6 as a positive control for SPR analysis. FABP ligand 6 exhibited a K_d_ of 1.26 μM, consistent with previous research and confirming data reliability (Figure 5C, left panel). In SPR sensorgrams, the maximum response signal intensity between Fabp5 and γ‐CEHC increased with increasing γ‐CEHC concentration, yielding a K_d_ of 5.24 μM (Figure 5C, right panel), indicating robust intermolecular affinity. Furthermore, the response curve declined after approximately 60 s, demonstrating reversible binding.

However, the specificity of binding of γ‐CEHC and Fabp5 remains uncharacterized. Additionally, the utility of other fatty acids that competitively inhibit this binding remains to be investigated. To validate the specificity of γ‐CEHC, we conducted additional SPR experiments. The results showed that the affinity of α‐CEHC for Fabp5 (*K*_d_ = 28.5 μM) was significantly lower than that of γ‐CEHCl (Figure 5D). Pre‐incubation with oleic acid markedly inhibited the binding between γ‐CEHC and Fabp5 (*K*_d_ = 74.2 μM) (Figure 5E). These data demonstrate that γ‐CEHC is a highly selective ligand for Fabp5. To investigate whether a Fabp5 antibody could antagonize this specific binding, we performed CETSA competition assays. Briefly, His‐tagged human recombinant Fabp5 protein was divided into three groups: control group (DMSO solvent only); γ‐CEHC group (30 μM γ‐CEHC), and γ‐CEHC + Fabp5 antibody group (pre‐incubated with 4 μg/mL anti‐Fabp5 antibody for 2 h to allow sufficient binding, followed by addition of 30 μM γ‐CEHC). Standard CETSA heating and Western blotting were performed. The results demonstrated that pre‐incubation with Fabp5 antibody significantly attenuated the γ‐CEHC‐induced decrease in Fabp5 thermal stability (Figure 5F). This finding confirms that the binding between γ‐CEHC and Fabp5 is specific and suggests that its binding epitope may overlap with or sterically hinder the antibody recognition site.

This study, using a multidimensional experimental system, confirms that γ‐CEHC is a highly selective natural inhibitor of Fabp5. This discovery establishes the molecular basis of the γ‐CEHC‐Fabp5 interaction and provides a theoretical foundation for its precise application in treating metabolic diseases and OP.

### 3.6 γ‐CEHC Restores M1/M2 Polarization Balance by Downregulating Fabp5, Promoting Osteoblast Differentiation and Suppressing Osteoclast Differentiation

**FIGURE 6:**
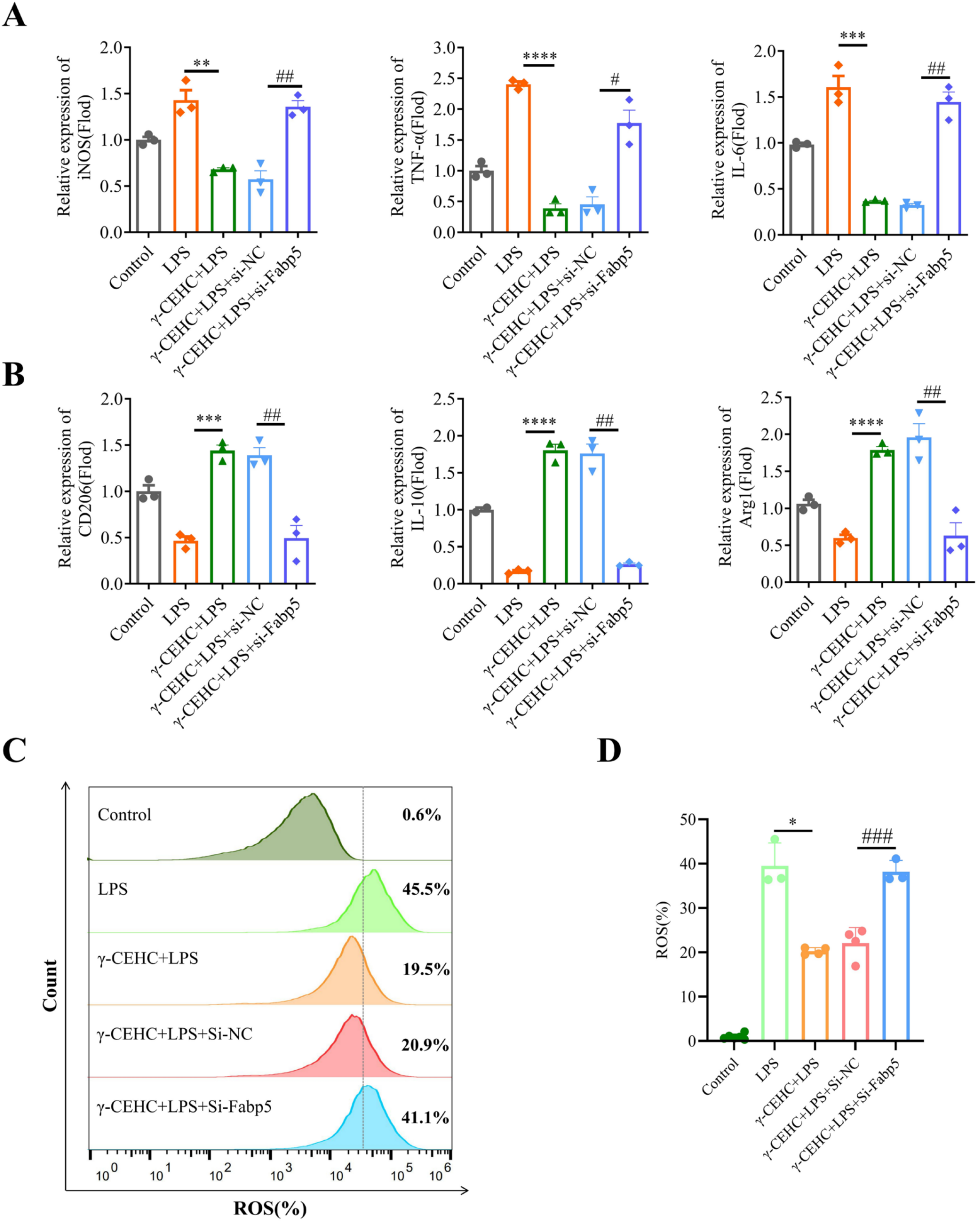
γ‐CEHC restores M1/M2 polarization balance by downregulating Fabp5. (A) Expression of M1 markers (*iNOS*, *TNF‐α*, and *IL‐6*) analyzed by qRT‐PCR. (B) Expression of M2 markers (*CD206*, *IL‐10*, and *Arg1*) analyzed by qRT‐PCR. (C) ROS levels labeled with fluorescent probes in each group as detected via flow cytometry. (D) Statistical results of ROS levels. All measurements are presented as means ± SD for three biological replicates (*p < 0.05, **p < 0.01, ***p < 0.001, ****p < 0.0001 indicates statistically significant differences of the γ‐CEHC treatment group relative to the LPS‐alone group; ^#^ *p* < 0.05, ^##^ *p* < 0.01, ^###^ *p* < 0.001 indicate statistically significant differences of the γ‐CEHC + LPS + empty vector group compared to the γ‐CEHC + LPS + si‐Fabp5 group).

Previous research has demonstrated that macrophage polarization directly affects both osteoblast and osteoclast differentiation [28], with Fabp5 playing a central role in regulating macrophage plasticity. Its deletion can simultaneously suppress pro‐inflammatory responses and enhance M2‐type anti‐inflammatory polarization through both PPARγ‐dependent and independent mechanisms. We examined the impact of γ‐CEHC on LPS‐induced ROS levels and macrophage polarization. We treated RAW264.7 cells with γ‐CEHC after inducing polarization. M1 and M2 surface marker expression analysis showed that, compared to the control group, LPS‐treated cells exhibited significantly elevated levels of M1 markers, including iNOS, TNF‐α, and IL‐6. In contrast, cells treated with γ‐CEHC displayed a substantial reduction in these M1 markers. Conversely, M2 markers, including CD206, IL‐10, and Arg1, were significantly downregulated by LPS, while γ‐CEHC treatment upregulated these markers (Figure 6A,B). Thus, γ‐CEHC promotes M2 macrophage polarization, and its anti‐inflammatory effects are attenuated by Fabp5 siRNA, demonstrating that Fabp5 is a direct target of γ‐CEHC. We assessed whether γ‐CEHC modulates LPS‐induced ROS production in macrophages. γ‐CEHC treatment significantly reduced ROS levels induced by LPS stimulation (Figure 6C,D), indicating a protective antioxidant effect.

Overall, γ‐CEHC restores the M1/M2 polarization balance by downregulating Fabp5, promoting osteoblast differentiation, and inhibiting osteoclast differentiation, demonstrating its potential as a therapeutic agent for OP.

## 4 Discussion

OP's global prevalence is estimated at 18.3%, with a nearly two‐fold higher rate in females (23.1%) than in males (11.7%) [29]. Even in developed countries, OP‐associated fractures impose a substantial economic burden, with costs reaching $17.9 billion annually in the United States and £4 billion in the United Kingdom [30]. An increasing body of evidence demonstrates that micronutrients, including minerals, trace elements, vitamins, and polyphenols, can affect the risk of developing OP [31, 32]. VE has been widely investigated for its bone‐protective properties. However, its activity varies considerably depending on its form [33]. VE metabolites exhibit distinct bioactivities and have significantly stronger anti‐inflammatory effects than unmetabolized VE compounds [5, 34]. We used TPP to generate a comprehensive interaction profile of γ‐CEHC‐binding proteins, providing a resource for future analyses.

Using TPP, we identified several potential target proteins of γ‐CEHC. Based on CETSA analysis over multiple species‐derived cell models, γ‐CEHC was found to specifically alter the thermal stability of Smarcal1, Fabp5, and Rhob. Smarcal1 (SWI/SNF‐associated, matrix‐associated, actin‐dependent chromatin regulatory factor subfamily A‐like protein 1) is a key molecular marker of Simcoe immune osseous dysplasia (SIOD) [35]. Mutations in both alleles are the cause of SIOD, affecting proteins associated with chromatin remodeling and DNA repair [36]. Patients with these mutations exhibit spondyloepiphyseal dysplasia, primarily affecting the spine, pelvis, femoral epiphyses, and pterygoid saddles, whereas the long bones of the hands and feet remain largely unaffected. Adolescents with this condition often develop secondary osteoporosis and hip joint issues, indicating that *Smarcal1* mutations disrupt bone metabolic homeostasis. Despite this, Smarcal1's utility as a diagnostic marker for SIOD is limited. Approximately 50% of suspected SIOD cases lack detectable *Smarcal1* mutations, and the skeletal phenotype does not indicate mutation presence, suggesting heterogeneity or other pathogenic mechanisms [37]. *Smarcal1* may influence skeletal development by regulating osteoblast differentiation and bone matrix metabolism; mutations can cause abnormal collagen metabolism or extracellular matrix defects, leading to characteristic skeletal lesions. Our study identifies Smarcal1 as a γ‐CEHC target protein that promotes osteoblast differentiation, offering a potential strategy for addressing hereditary bone diseases. Small Rho GTPases have been implicated in cell adhesion, motility, proliferation, survival, and inflammation [38, 39]. Rhob exhibits distinct expression patterns and biological functions compared to Rhoa and Rhoc. A recent study found that dexamethasone (Dex) induces Rhob expression at both mRNA and protein levels in the osteoblast‐derived osteosarcoma cell line MG‐63. The upregulation of *Rhob* mRNA by Dex occurs at the post‐transcriptional level, enhancing mRNA stability through the PI‐3 K/Akt and p38 mitogen‐activated protein kinase signaling pathways. Overexpression of *Rhob* in MG‐63 cells amplified Dex‐induced growth inhibition, while downregulation of *Rhob* via RNA interference attenuated this effect without impacting differentiation [40]. Furthermore, *Rhob* overexpression mimicked the impact of Dex on cell adhesion and migration, while interference with Rhob expression partially limited Dex‐induced pro‐adhesion and anti‐migration effects in MG‐63 cells. In conclusion, these findings demonstrate that Rhob is crucial for the pathological effects of Dex on osteoblastic growth and migration [40]. However, the role of Rhob in osteoclast differentiation remains unknown. This study validates that γ‐CEHC directly interacts with Rhob and regulates osteoclast function, providing the missing critical piece for elucidating the complete mechanism underlying its therapeutic effects against osteoporosis.

Fabp5 showed decreased stability in all human and murine cell lines, indicating it is a core target through which γ‐CEHC regulates bone metabolism. Fatty acid‐binding proteins (FABPs) are cytoplasmic proteins with a molecular weight of approximately 15 kDa, widely expressed in most mammalian tissues with specialized functions. This protein family is crucial for fatty acid transport and metabolism and is closely associated with metabolic disorders and abnormal cell proliferation [41]. As a key member of the FABP family, Fabp5 has received significant attention recently. Multiple studies have reported that aberrant expression of *Fabp5* under pathological conditions is associated with various diseases, including metabolic disorders [42], psoriasis [43], Alzheimer's disease [44], and malignant tumors [45], indicating its potential for clinical applications. Several small‐molecule inhibitors targeting FABP proteins have been developed as a result. However, most studies on FABP inhibition have focused on Fabp4, facing challenges such as nonspecificity, off‐target effects, and safety concerns. Our findings suggest that γ‐CEHC is a highly selective natural inhibitor of Fabp5, serving as an effective strategy for treating diseases associated with FABP proteins. Breakthrough research on the molecular functions of immune factors and inflammatory cytokines has provided evidence that Fabp5 may participate in cytokine production by regulating cellular lipid metabolism and signaling pathways [46]. Previous studies have reported that the expression of inflammatory genes (such as *COX2* and *IL‐6*) is suppressed in Fabp5‐deficient macrophages [47]. Additionally, compared with wild‐type mice, *Fabp5* knockout mice exhibit higher mRNA levels of anti‐inflammatory cytokines, including *IL‐10*, *arginase*, *YM1*, and *Fizz‐1*, in the liver [48]. These findings indicate that Fabp5 is a key regulator of pro‐inflammatory responses and closely interacts with multiple cytokines. The homeostasis of macrophage phenotypes (M1/M2) is a critical component of inflammation [49]. Some researchers have proposed that Fabp5 deficiency may cause elevated levels of M2‐related cytokine expression [48], aligning with our findings. Peroxisome proliferator‐activated receptors (PPARs) are a superfamily of nuclear receptor transcription factors, comprising three subtypes: PPARα, PPARβ/δ, and PPARγ [50]. Recent studies have robustly confirmed direct interactions between Fabp5 and the PPAR family [51, 52]. Fabp5 deficiency suppresses pro‐inflammatory signaling and promotes M2‐type anti‐inflammatory polarization through both PPARγ‐dependent and independent pathways [51]. Notably, PPARβ/δ signaling also plays a pivotal role in regulating macrophage polarization, with studies indicating that its absence significantly impairs the IL‐4‐induced M2 polarization process. S‐glutathionylation at Cys127 of Fabp5 participates in the interaction between Fabp5 and PPARβ/δ, activates downstream target genes of PPARβ/δ, and thereby inhibits LPS‐induced inflammatory responses in macrophages [53]. However, it remains unclear whether γ‐CEHC‐bound Fabp5 delivers natural ligands to PPARγ with reduced efficiency, regulating the transcription of PPARγ target genes involved in macrophage polarization, or whether γ‐CEHC binding mimics or interferes with the natural regulation of S‐glutathionylation participating in Fabp5‐PPARβ/δ interaction and anti‐inflammatory effects. The molecular mechanisms involved will inform our future research.

This study is the first to reveal γ‐CEHC‐induced dynamic proteomic changes, identify and validate potential γ‐CEHC target proteins, and explore its regulatory effects on osteogenic and osteoclastic differentiation. These results offer novel insights into OP treatment. Our findings suggest that γ‐CEHC exerts antioxidant and anti‐inflammatory effects in osteoblasts and osteoclasts, helping to clarify the link between γ‐CEHC and the improvement of OP. This work lays the foundation for future research on the targets and mechanisms underlying γ‐CEHC's anti‐osteoporotic effects.

However, this study has certain limitations. While we validated the effects of γ‐CEHC across multiple cell models, including murine RAW264.7, MC3T3‐E1, and human THP‐1 and hFOB1.19 cells, it is vital to acknowledge the limitations of cell line models in reflecting the in vivo pathophysiological environment. For example, cell lines may lose a portion of their original phenotypes during long‐term culture and cannot fully simulate the complex intercellular communication and microenvironmental regulation in vivo. We incorporated animal experiments and cell‐based validation across multiple species to enhance the reliability of our conclusions at numerous levels. Future studies should consider introducing primary cells or organoid models to validate the mechanisms of γ‐CEHC under conditions more closely resembling the physiological environment.

Overall, γ‐CEHC, an endogenous Fabp5 inhibitor, integrates metabolic regulation, anti‐inflammatory activity, and bone protection, making it a compelling candidate molecule for developing innovative “one‐target‐multiple‐diseases” therapeutic agents. Its natural origin may overcome potential toxicity risks associated with synthetic inhibitors, demonstrating broad avenues for combination therapies targeting OP, metabolic syndromes, and tumor immunology.

## Funding

The author(s) declare financial support was received for the research, authorship, and/or publication of this article. The present study was supported by the Natural Science Foundation of Beijing (7264289), National Key R&D Program of China, MOST (Grant No. 2023YFC2509900), Project supported by Beijing Jishuitan Research Funding (Grant No. KYYC202301), and National Key R&D Program of China, MOST (Grant No. 2024YFC3044700).

## Conflicts of Interest

The authors declare no conflicts of interest.

## Supporting information


**Figure S1:** Workflow of quantitative analysis of γ‐CEHC‐interacting proteins in MC3T3‐E1 and RAW264.7 cell lysates via TPP (created with https://BioRender.com).

**Figure S2:** Quality control analysis of the database search and mass spectrometry findings based on the thermal proteome profiling in MC3T3‐E1 cells. (A) Overview of protein identification. (B) Peptide length distribution. (C) Molecular weight distribution of identified proteins. (D) Protein sequence coverage distribution. (E) GO enrichment bar chart demonstrating the relative expression of DEPs in different pathways.

**Figure S3:** Quality control of the database search and mass spectrometry results based on thermal proteome profiling in RAW264.7 cells. (A) Overview of protein identification. (B) Peptide length distribution. (C) Molecular weight distribution of identified proteins. (D) Protein sequence coverage distribution. (E) GO enrichment bar chart presenting relative expression of DEPs in different pathways.

**Table S1:** Sequences of primers for qRT‐PCR.

**Table S2:** List of proteins detected by quantitative proteome analysis in MC3T3‐E1 cells.

**Table S3:** List of proteins with modified thermal stability following γ‐CEHC treatment in MC3T3‐E1 cells.

**Table S4:** List of proteins identified by quantitative proteome analysis in RAW264.7 cells.

**Table S5:** List of proteins with modified thermal stability following γ‐CEHC treatment in RAW264.7 cells.

## Data Availability

The data that support the findings of this study are available on request from the corresponding author. The data are not publicly available due to privacy or ethical restrictions.
