# Supplementary figures and images for: Fabp5 Is the Key Regulator Mediating γ‐CEHC Differentiation in Osteoblasts and Osteoclasts
Source: Biofactors. 2026 Jan 19;52(1):e70079. doi: 10.1002/biof.70079 (PMC12813964; doi:10.1002/biof.70079)

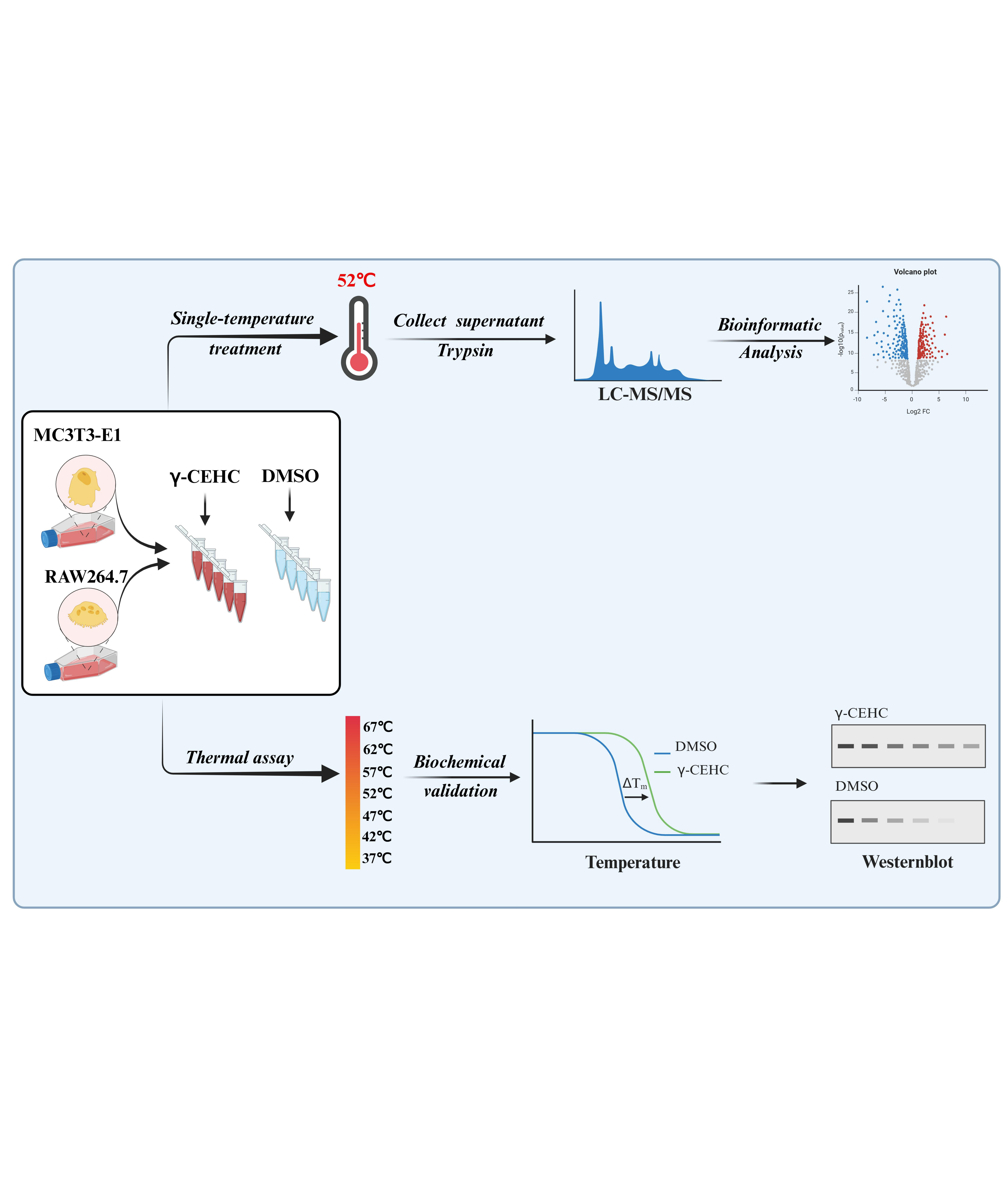

Supplement: Supplementary file 1 — Figure S1: Workflow of quantitative analysis of γ‐CEHC‐interacting proteins in MC3T3‐E1 and RAW264.7 cell lysates via TPP (created with https://BioRender.com). [file BIOF-52-0-s008.jpg]

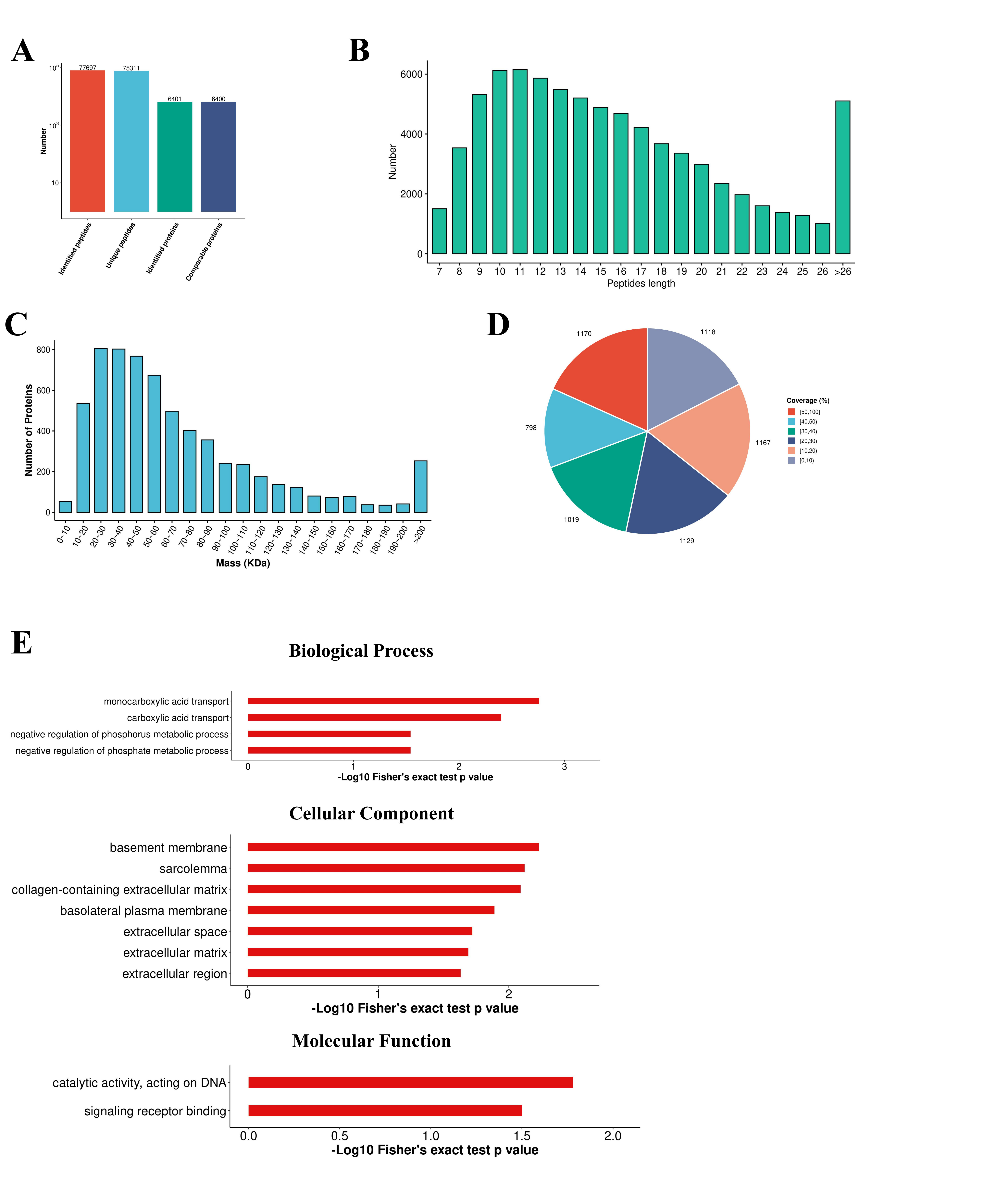

Supplement: Supplementary file 2 — Figure S2: Quality control analysis of the database search and mass spectrometry findings based on the thermal proteome profiling in MC3T3‐E1 cells. (A) Overview of protein identification. (B) Peptide length distribution. (C) Molecular weight distribution of identified proteins. (D) Protein sequence coverage distribution. (E) GO enrichment bar chart demonstrating the relative expression of DEPs in different pathways. [file BIOF-52-0-s006.jpg]

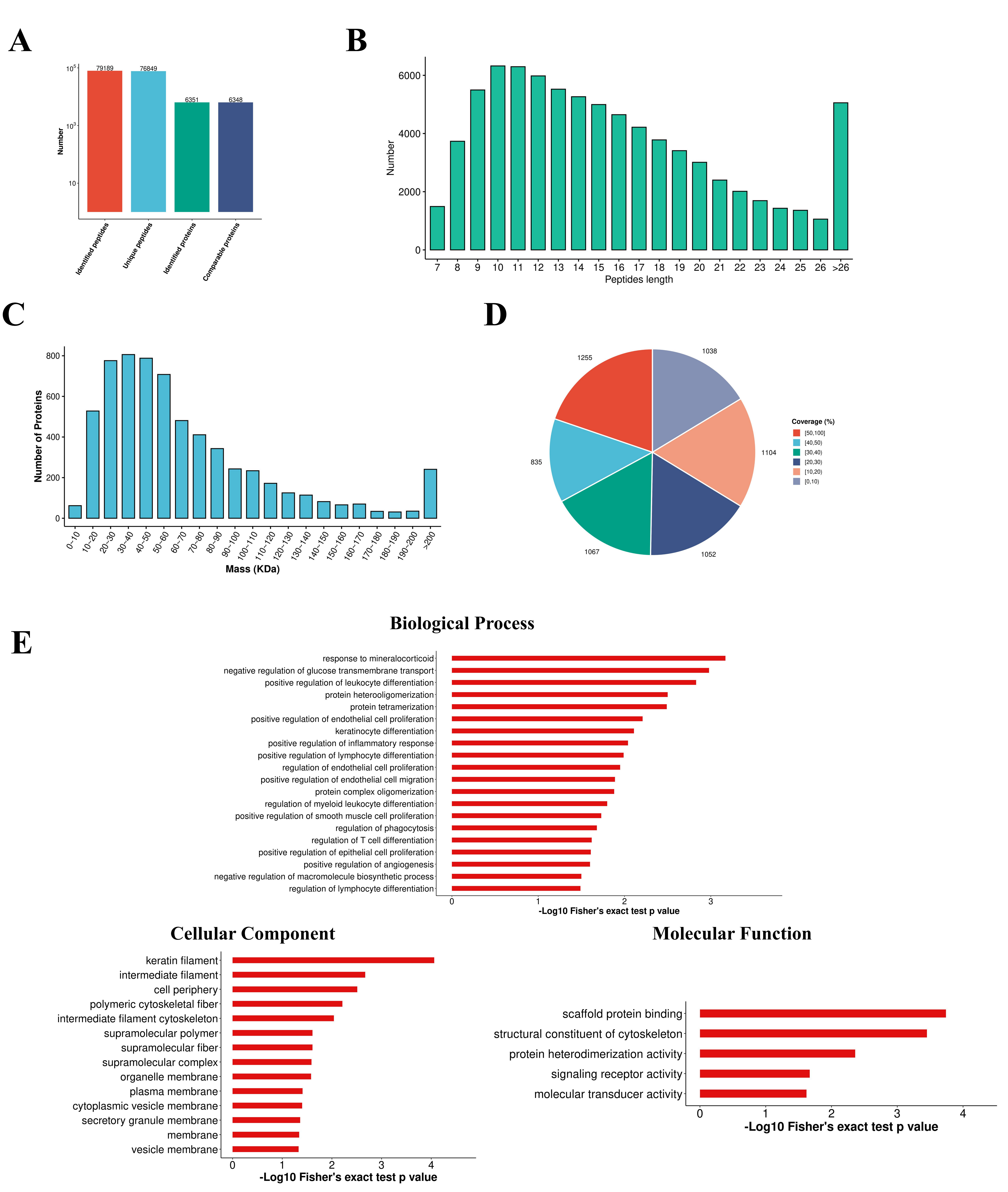

Supplement: Supplementary file 3 — Figure S3: Quality control of the database search and mass spectrometry results based on thermal proteome profiling in RAW264.7 cells. (A) Overview of protein identification. (B) Peptide length distribution. (C) Molecular weight distribution of identified proteins. (D) Protein sequence coverage distribution. (E) GO enrichment bar chart presenting relative expression of DEPs in different pathways. [file BIOF-52-0-s002.jpg]
