# Supplementary material for: Fabp5 Is the Key Regulator Mediating γ‐CEHC Differentiation in Osteoblasts and Osteoclasts
Source: Biofactors. 2026 Jan 19;52(1):e70079. doi: 10.1002/biof.70079 (PMC12813964; doi:10.1002/biof.70079)
Supplement: Supplementary file 4 — Table S1: Sequences of primers for qRT‐PCR. [file BIOF-52-0-s001.docx]

Supplementary Table 1. Sequence of cell primers for qRT-PCR

| **Gene** | **Forward (5’-3’)** | **Reverse (5’-3’)** |
| --- | --- | --- |
| iNOS | CCAGCTAGCCAAAGTCACCAT | GTCTCGGAGCCATACAGGATT |
| TNF-α | GAGCAAGCCCTGGTATG | CGGGCCGATGAGATGATCTCTCG |
| IL-6 | TGCAATAACCCCTGACC | ATTTGCCGAAGCCCG |
| CD206 | TGATACCTGCGACAGTAAACGA | CTTGCAGTATGTCTCCGCTTC |
| IL-10 | GCCAAGCCTTGTCTGAGATGATCC | AATCGATGACAGCGCCGTAGC |
| Arg1 | GTGGAAACTTGCATGGACAAC | AATCCTGGCACATCGGGAATC |
| GAPDH | GACTCATGACCACAGTCCATGC | AGAGGCAGGGATGATGTTCTG |
| Nfatc1 | GGAGAGTCCGAGAATCGAGAT | TTGCAGCTAGGAAGTACGTCT |
| C-fos | CGGGTTTCAACGCCGACTA | TTGGCACTAGAGACGGACAGA |
| Ctsk | GAAGAAGACTCACCAGAAGCAG | TCCAGGTTATGGGCAGAGATT |
| Acp5 | CACTCCCACCCTGAGATTTGT | CATCGTCTGCACGGTTCTG |
| Src | GAACCCGAGAGGGACCTTC | GAGGCAGTAGGCACCTTTTGT |
| Ocn | TCTGACCTCACAGATGCCAAG | AGGGTTAAGCTCACACTGCT |
| Runx2 | CATGGCCGGGAATGA TGAG | TGTGAAGACCGTTATGGTCAAAGTG |
| Col1 | GCTCCTCTTAGGGGCCACT | CCACGTCTCACCATTGGGG |
|  |  |  |
|  |  |  |
|  |  |  |
